# Antioxidant Activity of Blueberry (*Vaccinium* spp.) Cultivar Leaves: Differences across the Vegetative Stage and the Application of Near Infrared Spectroscopy

**DOI:** 10.3390/molecules24213900

**Published:** 2019-10-29

**Authors:** Ricardo N.M.J. Páscoa, Maria João Gomes, Clara Sousa

**Affiliations:** LAQV/REQUIMTE, Departamento de Ciências Químicas, Faculdade de Farmácia, Universidade do Porto, Rua Jorge Viterbo Ferreira, 228, 4050-313 Porto, Portugalup201304726@ff.up.pt (M.J.G.)

**Keywords:** *Vaccinium ashei*, *Vaccinium corymbosum*, phenolics, flavonoids, chemometrics

## Abstract

Blueberries production has increased in the last few years boosted somehow by the World Health Organization (WHO) recommendations for a healthier nutrition and their recognized potential to treat several diseases. The production increase lead to high amounts of discarded leaves that could be very valuable. In this context, the antioxidant activity of *Vaccinium* spp. leaves, by means of the total phenolic (TPC) and flavonoid (TFC) content and total antioxidant capacity (TAC) was determined. Adult leaves of twenty-seven *Vaccinium* cultivars collected in three geographic regions and three seasons of the year were included. The antioxidant activity was additionally estimated with near infrared (NIR) spectroscopy and data transferability across the regions and seasons was evaluated. The TPC, TFC and TAC ranged from 39.6–272.8 mg gallic acid, 41.2–269.1 mg catechin and 22.6–124.8 mM Trolox per g of dry leaf, respectively. Globally through the seasons, the higher values of the three parameters were obtained in December. Regarding the geographic region, region A provided the cultivars with the higher antioxidant content. Titan was the cultivar with higher TPC and TAC and Misty the one with the higher TFC. NIR spectroscopy combined with the partial least squares analysis was able to successfully predict the antioxidant activity with coefficients of determination and range error ratios ranging from 0.84–0.99 and 11.2–26.8. Despite some identified limitations on data transferability, NIR spectroscopy proved to be a reliable, low cost and quick method to predict the antioxidant activity of the considered cultivar leaves.

## 1. Introduction

Blueberries are considered a super food mainly due to its high content of antioxidant compounds as anthocyanins, flavonols and chlorogenic acid [1]. They are recognized as possessing a strong potential in the treatment of cancer, diabetes, cardiovascular diseases and dementia [2,3,4]. Such evidences, together with the recommendations from WHO to increase fruit and vegetables consumption [5], boosted the interest in blueberries and their production strongly grown in the last few years. With such increase, large amounts of *Vaccinium* leaves are also being produced and discarded. Some studies pointed that leaves possess similar or even higher antioxidant capacity than blueberries themselves making them an interesting resource [6,7]. Indeed, the antioxidant potential of some *Vaccinium* spp leaves, mainly of *Vaccinium ashei* cultivars [8,9,10], have already been determined. Some of these studies also made some considerations about the influence of the harvest season in their antioxidant activity [9,10]. Nevertheless, *Vaccinium corymbosum* leaves have been barely explored. Ehlenfeldt & Prior [6] and Wang et al [11] determined the antioxidant activity of several *V. corymbosum* leaves but with no reference to the harvest season. Three additional studies referred and compared the results among the harvest season but only included 1 to 3 *V. corymbosum* cultivars [12,13,14]. *V. corymbosum* is one of the most commercially relevant blueberries species among the world. Also, due to the differences noticed among cultivars, harvest seasons and the same cultivar grown in different soils, their characterization is of upmost relevance. Additional knowledge over such plants will allow each producer to select the most profitable cultivar. Some of the standard methods used in the determination of the antioxidant activity of berries and leaves [15,16] are laborious, time consuming and requires the use of several reagents making their determination cumbersome. NIR spectroscopy coupled with appropriate chemometric methods have been successfully applied to predict more of less complex food or food systems properties, including the prediction of the antioxidant activity. Magalhães and colleagues [17] predicted the quantity of three bioactive phenolics and three methylxanthines in spent coffee grounds with a coefficient of determination (R^2^) ranging from 0.71–0.95 for the prediction set samples. Páscoa et al. [18] used NIR spectroscopy to predict TPC and TAC of red grape pomace attesting the suitability of this based infrared technique to estimate the antioxidant activity of food matrices. Several other works were published reporting the usefulness of this technique to predict the antioxidant capacity of distinct food matrices [19,20,21,22,23] with distinct degrees of success. However, NIR spectroscopy was never used to characterize blueberries leaves.

The present work was developed in this context and aims: (I) the determination of the antioxidant activity of 27 distinct cultivar leaves, mostly of *V. corymbosum*; (II) to infer about its variation across the harvest seasons and regions and (III) to propose a quick, low-cost and reagent free technique, near infrared spectroscopy (NIR spectroscopy) to predict their antioxidant activity.

## 2. Results

### 2.1. Cultivars Antioxidant Activity

**Figure 1:**
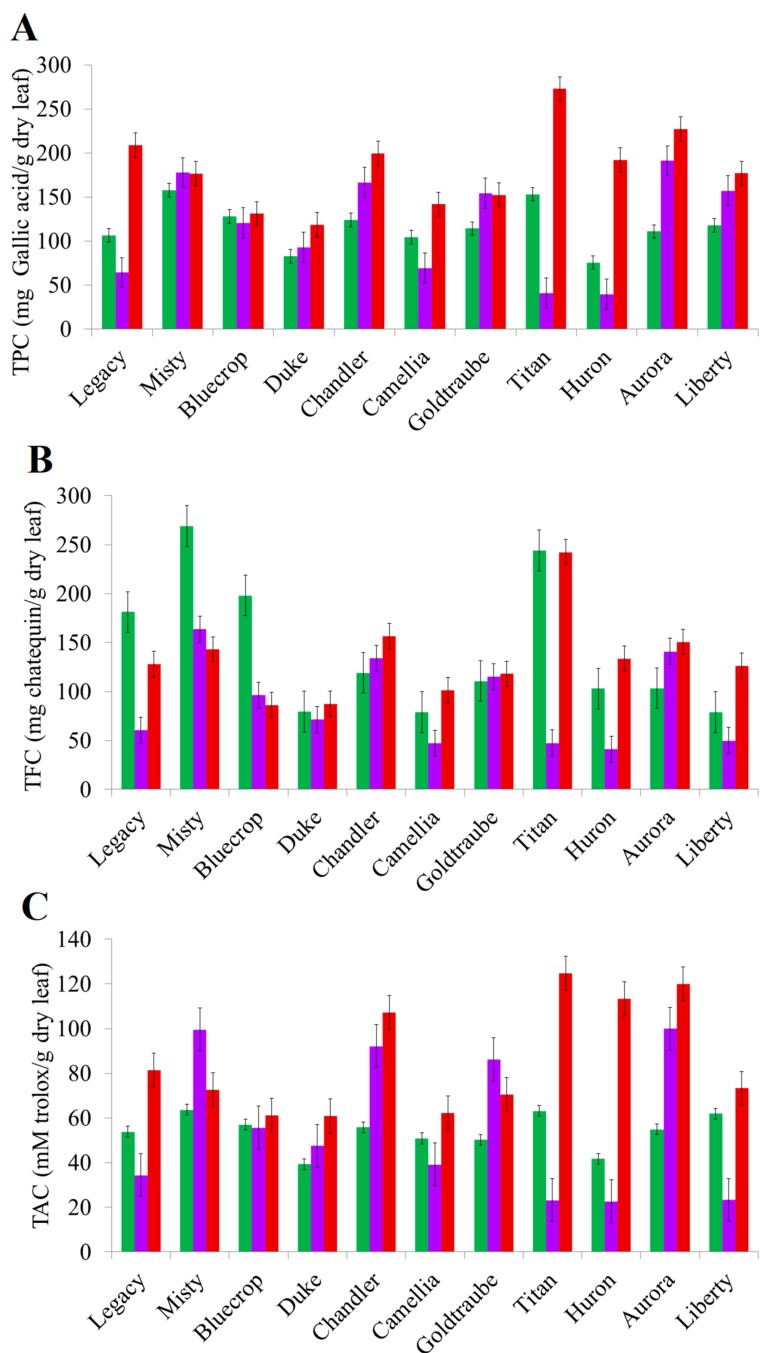
Total Phenolic Content (**A**); Total Flavonoid Content (**B**) and Total Antioxidant Capacity (**C**) of the hydroalcoholic extracts from cultivars collected in the three considered seasons of the year. Legend: █ May; █ September; █ December.

The antioxidant activity was determined for 27 distinct *Vaccinium* cultivar leaves harvest in three distinct geographic regions (A- Northern Coast; B- Northern Inland and C- South Inland) across Portugal and during three seasons (Spring- May; Fall- September and Winter- December). The cultivar antioxidant activity was evaluated by means of the total phenolic content (TPC), total flavonoid content (TFC) and total antioxidant capacity (TAC). Hydroalcoholic air-dried milled leaves extracts were used to obtain the three parameters (For fully details please see Section 4- Material and Methods). A detailed statistical analysis was performed (Tables S1–S18 of the Supplementary Material) including the comparisons of the TPC, TFC and TAC among cultivars of each geographic region and season and among each cultivar across the three seasons. The analysis could be used to support the interpretation of Figure 1 and Table 1, Table 2 and Table 3.

#### 2.1.1. Total Phenolic Content (TPC)

The results obtained for the TPC of all the *Vaccinium* cultivar leaves extracts are presented in Table 1. Among all the harvest seasons and regions, the highest TPC was found in ‘Titan’ extracts (272.8 ± 4.0 mg gallic acid/g dry leaf) from winter leaves while the lowest TPC was found in ‘Huron’ extracts (39.6 ± 1.8 mg gallic acid/g dry leaf) from fall leaves. Considering each harvest season *per se*, the TPC values largely varied among the cultivars. The values are different even within the same season when leaves are collected from two different geographic regions. Comparing the TPC of the cultivars harvested in the three seasons (Figure 1A) it is of remark that the higher TPC was always found in the winter leaves (*p* < 0.05 for all cultivars) except for ‘Misty’ and ‘Goldtraube’ cultivars for which the TPC content is similar (*p* > 0.05) and lower (*p* < 0.05) than those determined in fall leaves, respectively.

#### 2.1.2. Total Flavonoid Content (TFC)

Results for the TFC of the cultivar leaves extracts are presented in Table 2. The highest TFC was determined for ‘Misty’ extracts (269.1 ± 7.4 mg catechin/g dry leaf) from spring leaves and the lowest for ‘Huron’ (41.2 ± 4.8 mg catechin/g dry leaf) from fall leaves. Similarly, to the observed for the TPC, the TFC largely varies among cultivars collected in the three seasons and geographic regions. The relative TFC of the different cultivars was also not the same across the seasons. The TFC of the cultivars harvested in the three seasons (Figure 1B) presented higher fluctuations than the TPC being the higher values reported for winter season leaves except for ‘Legacy’, ‘Misty’ and ‘Bluecrop’ cultivars which presented higher TFC for spring leaves (*p* < 0.05 for the three cultivars) extracts.

#### 2.1.3. Total Antioxidant Capacity (TAC)

Results for the TAC of the cultivar leaves extracts are presented in Table 3. ‘Titan’ winter leaves extracts showed the highest TAC (124.8 ± 2.5 mM Trolox/g dry leaf) while the lowest values were found in ‘Huron’ fall leaves extracts (22.6 ± 0.7 mM Trolox/g dry leaf). Regarding each season individually, spring leaves extracts presented more similar TAC values between cultivars while the TAC values obtained for fall and winter leaves were different. TAC values across the seasons (Figure 1C) were found to be higher (*p* < 0.05 for all the cultivars) in winter leaf extracts except for ‘Misty’ and ‘Goldtraube’ for which the TAC of fall leaves extracts were higher (*p* < 0.05 for both cultivars).

### 2.2. Antioxidant Activity Prediction by NIR Spectroscopy

The antioxidant activity by means of the TPC, TFC and TAC was estimated through the air dried leaves near infrared spectra for the previously defined six data sets (Section 4.2), considering each data set independent. The NIR technique is known as a “fingerprint” technique since it captures the chemical information related with the hydrogen atom, namely C-H, O-H, N-H and S-H bonds, present on the samples. In this sense, the existing chemical compounds in *Vaccinium* cultivar leaves that possess antioxidant capacity contribute to NIR spectra. The figures of merit obtained from the best partial least squares (PLS) models were presented in Table 4. Globally, the best results were always obtained considering the spectral regions from 6315–5390 cm^−1^ and 4925–4073 cm^−1^ (for additional details please see Figure S1) with five to nine latent variables (LVs). The coefficients of the determinations were also very satisfactory, ranging from 0.86 to 0.99 and the range error ratios (RER) values from 11.2 to 25.5. Regarding the estimates of each parameter individually, the lowest root mean square errors of calibration, cross validation and prediction and higher coefficients of determination were obtained for data sets 4 (leaves collected in spring in a single region: RA) to 6 (leaves collected in winter: RA). Data set 1 (all the leaves: spring/fall/winter, RA/RB/RC) and 2 (leaves collected from a single region: spring/fall/winter, RA) originated poorer results. Regarding data set 3 (leaves collected in spring in three regions: RA/RB/RC), the TPC and TAC estimates were satisfactory as those achieved with data sets 4 to 6 but the models performance of the TFC estimates were similar to the results obtained with data set 1 and 2.

## 3. Discussion

This study encompassed a multidisciplinary approach to explore the antioxidant activities of several *V. corymbosum* and *V. ashei* cultivar leaves, collected in distinct geographic regions and seasons, and to propose a quick and low cost method to predict the antioxidant activities. The cultivars included in the study were select in order to cover the ones more commonly explored commercially. The geographic regions were chosen based on the existent cultivars and in order to be distant enough to provide different edaphoclimatic conditions. There are few previous works reporting the antioxidant activities of *Vaccinium* cultivars and most of these works did not considered different cultivars, different harvest seasons and geographic regions. Li et al. [8] and Zhu et al. [9] analysed *V. ashei* leaves without any reference to the cultivars included in their studies. According to the literature [10,11] and the results obtained in this work, the antioxidant activity is highly dependent of the cultivar even within the same species. Other studies did not consider the differences among the leaves harvest season (Kim & Um 2011; Wang et al. 2015) and those who did it included only a few cultivars, all collected in the same geographic region [13,14]. Regarding *V. ashei* cultivars included in our study (‘Powderblue’, ‘Ochlochonee’ and ‘Titan’), lower TPC, TFC and TAC values were found when compared to the *V. ashei* leaves analysed by Zhu and co-workers (2013) [9]. However, these authors did not specify the cultivars of *V. ashei* used which could justify the differences found. Besides, Zhu and co-workers used boiling water as the extraction solvent which also prevents reliable comparisons with our results. It is known that the solvent plays a fundamental role in the extraction process [14]. Cezarotto et al. [10] determined the TPC of ‘Powderblue’ using the extraction solvent of this work (water:ethanol) in leaves collected in December and March. Their results from March leaves were more similar to those obtain from May leaves in this work; however, they found in December leaves a much lower TPC. It should be noted that in December most of the *Vaccinium* plants already have less and poor quality leaves depending on the edaphoclimatic conditions of the year/region which could contribute to the discrepancy of the results. Regarding the *V. corymbosum* cultivars common to literature and this work, Venskutonis et al. [14] found for ‘Bluecrop’ in three consecutive months (June/July/August) a TPC ranging from 40 to 200 mg GAE/g dry leaf and a TAC from 0.08 to 0.14 mg/mL of Trolox being the results highly dependent on the extraction solvent (acetone and methanol:water). The results herein obtained, even with a different extraction solvent, compared quite good with those of Venskutonis and colleagues considering the May leaves (of note, the TAC value obtained in this work for ‘Bluecrop’ was around 0.98 mg/mL of Trolox). Piljac-Žegarac and co-workers (2009) [24] also determined the TPC of ‘Bluecrop’ in leaves collected at late summer, with boiling water as extraction solvent (from 198 to 394 mg/L depending on the extraction time) being the results similar to those achieved in this work (around 291 mg/L). Kim and Um [12] also determined the TPC (1 mg/mL) of ‘Duke’ cultivar with methanol:acetic acid:water (25:1:24; *v*/*v*/*v*) as extraction solvent from leaves collected in July being their result higher than that obtained in this work (around 0.22 mg/mL for May leaves). Routray and Orsat (2014) [13] determined the TPC of ‘Elliot’ and reported values from 106.1 to 155.8 mg GAE/g dry leaf depending on the collecting month, being the higher value obtained from October leaves. Our ‘Elliot’ cultivar leaves were collected only in December and possess a higher TPC (199.0 mg GAE/g dry leaf). No further studies were found focusing the remaining *Vaccinium* cultivars included in our work which prevents additional comparisons. In general, *Vaccinium* cultivars winter leaves included in this work possessed higher antioxidant potential which was reflected in their higher TPC, TFC and TAC emphasizing the relation between the antioxidant compounds present in the leaves and the vegetative stage of the plant. Regarding the existing literature, it is known that plants produce more antioxidant compounds to answer to dynamic changes in the environment [25]. In this sense, the plants in the winter are exposed to lower temperatures which may trigger the production of antioxidant compounds. Further studies are needed to clarify the reasons under fluctuations of these compounds across the harvest seasons. Moreover, it can be stated that the geographic region and the plant cultivar have a strong impact on the amount of the antioxidant compounds. From the *Vaccinium* cultivars analyzed, “Titan” winter leaves presented the highest amount of TPC and TAC while for TFC it was the third cultivar. On the other hand, “Huron” fall leaves presented the lowest amount of TPC, TFC and TAC. This makes sense since the parameters analyzed herein are indirectly related [26]. When comparing the results obtained from the different geographic regions with the same cultivars, it is evident that region A (Northern coast) presented the highest amount of TPC (except for “Duke” and “Camellia” cultivars which present similar TPC content in both regions; *p* > 0.05), higher TFC (except for “Duke” cultivar which present lower TFC content in region A; *p <* 0.05) and TAC (except for “Duke” and “Powderblue” cultivars for which the content is similar in both regions; *p* > 0.05). This indicates that the geographic regions have a strong impact on *Vaccinium* plants. Another interesting finding from the geographic region comparison was that “Duke” cultivar does not show a significant difference between TPC and TAC parameters (*p* > 0.05) at both regions. This could point to a good capacity of adaption of this cultivar to different environments. Altogether, these results highlight the need of a meticulous cultivar choice depending on the farmers’ main targets. The infrared based estimates of TPC, TFC and TAC were globally satisfactory in terms of root mean square errors and coefficients of determination (Table 4). Also, the range error ratios (RER) of the developed PLS models fits well with the guidelines for near-infrared models development and maintenance. When RER ≥ 10, the models are acceptable for calibration for quality control and when RER ≥ 15, the models are very good for calibration for quantification [27]. Indeed, all of the herein developed PLS models had RER ≥ 10 being most of them considered proper for quantification proposes (RER ≥ 15). Globally, the best results were achieved with data sets 4 to 6 which encompassed spectra from a single region and/or season. It seems that leaves spectra from the remaining sets (including leaves from more than one season and/or geographic region) contain some masking information which decreases the performance of the PLS models. This information should be considered when using such models which need to be developed and further validated within well-defined parameters. The worst PLS models for most of the parameters were found when using leaves collected in the spring from all the regions (Set 3). Also, comparing the PLS models results considering only a single region but harvest season (Set 4, 5 and 6), the worst results were obtained for the leaves collected in the spring. The results herein obtained reflect the accuracy and suitability of NIR spectroscopy for determining these parameters even when considering all the leaves. In general, the TPC, TFC and TAC NIR spectroscopy predictions obtained in this work compared quite well with previous published studies on antioxidant properties and/or compounds being in some cases better [17,19,28,29]. Lu and collaborators [28] estimated the TPC of onion and shallots with a coefficient of prediction of 0.97 and 0.95 for the calibration and cross validation samples, respectively. Despite the high variation associated to the set samples considered, the coefficients of prediction obtained in this work for the same parameters ranging from 0.91–0.99 and 0.88–0.98. Páscoa et al. [29] estimated the TPC, TFC and TAC for Camellia japonica cultivars leaves extracts and obtained coefficients of prediction from 0.93–0.95 for the validation samples which were very similar to those found in this work. However, the RER values herein obtained were, in general, more satisfactory than those obtained by Páscoa and collaborators [29]. The results obtained in this work contributed (i) to increase the knowledge about the antioxidant activities of a large number of *Vaccinium* cultivars plants which are the main sources of the blueberries consumed around the world; (ii) to provide some information about the variations of the antioxidant activities across the seasons and geographic regions; (iii) to prove the ability of NIR spectroscopy combined with chemometrics to predict the antioxidant activity of the leaves and additionally (iv) to better understand the advantages and/or limitations of developing calibration chemometric models for crosswise application through seasons and geographic regions.

## 4. Material and Methods

### 4.1. Reagents and Solutions

Ultrapure water and ethanol absolute pro analysis were used for the preparation of all solutions and leaves extracts. Trolox ((±)-6-hydroxy-2,5,7,8-tetramethylchromane-2-carboxylic acid), 2,2′-Azino-bis(3-ethylbenzothiazoline-6-sulfonic acid) (ABTS) diammonium salt, sodium carbonate decahydrate, sodium nitrite, sodium hydroxide and gallic acid were obtained from Fluka (Buchs, Switzerland). Folin-Ciocalteu reagent, (+)-catechin hydrate, sodium acetate trihydrate, aluminium chloride hexahydrate, and potassium persulfate were obtained from Sigma-Aldrich (St. Louis, MO, USA). The total phenolic content (TPC) determination involved the preparation of 3:10 (*v*/*v*) in water of Folin-Ciocalteu reagent, 24.3% (*w*/*v*) of Na_2_CO_3_·10 H_2_O and a stock solution of 100 mg∙L^−1^ of gallic acid. The total flavonoid content (TFC) determination involved the preparation of 6 g∙L^−1^ NaNO_2,_ 0.8 M NaOH, and 22 g∙L^−1^ AlCl_3_ · 6 H_2_0 solutions as well as a stock solution of 250 mg∙L^−1^ of (+)-catechin prepared daily. For the total antioxidant capacity (TAC) determination, an ABTS^●+^ radical solution was prepared daily with equal volumes of 14 mM of ABTS stock solution and 7 mM of potassium persulfate and kept for 12–16 h before being used at dark conditions and a stock solution of 1.0 mM of Trolox.

### 4.2. Vaccinium Cultivar Leaves

Adult leaves fully exposed to sunlight of thirty-five plants (P = 35) from twenty-seven distinct cultivars (C = 27) belonging to *Vaccinium ashei* (n = 3) and *Vaccinium corymbosum* (n = 24) were included in this work. Leaves were collected during the year of 2017 in three distinct regions of Portugal: two in the north (RA and RB) and one in the south (RC) of Portugal. Leaves from north region A (RA) were also collected in three distinct seasons (spring: 30th of May, fall: 1st of September and winter: 1st of December) encompassing different development stages of the plants (presence or absence of berries). Details about the cultivars, collecting region, season and plant stage were presented in Table 5. Twenty leaves per plant were harvested and air-dried (T~20 °C) protected from the daylight to constant weight and further pulverized and sieved for all the subsequent analysis after collection. The antioxidant activity, by means of the TPC, TFC and TAC, of all the leaves was determined from their hydroalcoholic extracts as described in Section 4.3. Regarding infrared data analysis, leaves were grouped into six data sets: set 1- all the leaves (spring/fall/winter, RA/RB/RC); set 2- leaves collected from a single region (spring/fall/winter, RA); set 3- leaves collected in spring in three regions (RA/RB/RC); set 4- leaves collected in spring in a single region (RA); set 5- leaves collected in fall (RA) and set 6- leaves collected in winter (RA).

### 4.3. Antioxidant Activity

Air-dried milled *Vaccinium* leaves (50 mg) were extracted in 20 mL of an ethanolic/water (50/50, *v*/*v*) solution for 240 min in an orbital shaker at 300 rpm. After the extraction, samples were centrifuged at 9200× *g* for 2 min (Jouan Bra1 Multifunction Centrifuge, Thermo Electronic) and the clear hydroalcoholic solution was appropriately diluted with water for assessment of TPC, TFC and TAC (50, 10 and 50 times, respectively). The extraction time was selected by the measurement of the amount of extracted phenolics (TPC method) after 60, 120, 180, 240, and 300 min. The extraction yield after 240 min was not statistically different (*p* > 0.05) from those determined at longer extraction times. Extracts were obtained in duplicate in two distinct days.

#### 4.3.1. Total Phenolic Content (TPC)

Folin-Ciocalteu assay was performed in a 96-well microplate as described in Magalhães et al [16]. Briefly, 150 μL of diluted extracts (1:50), 50 μL of Folin-Ciocalteu reagent (3:10, *v*/*v*) and 100 μL of carbonate buffer solution (9% (*w*/*v*) were consecutively added to each well. The reduction at alkaline pH of the Folin- Ciocalteu reagent by leaves phenolics was monitored at 760 mM during 120 min. Gallic acid standard solutions were used for calibration. The intrinsic absorption of samples was evaluated with HCl (0.6 M) instead of the Folin-Ciocalteu reagent. TPC was expressed as gallic acid equivalents (mg gallic acid/g of sample) and each extract was analysed in quadruplicate.

#### 4.3.2. Total flavonoid Content (TFC)

The total flavonoid content (TFC) of the diluted hydroalcoholic extracts (1:10) was determined through their absorbance at 510 mM [15]. In a 96-well plate, 75 μL of the diluted extract and 75 μL of a NaNO_2_ (6 g∙L^−1^) solution were placed in each well. After 5 min, 75 μL of an AlCl_3_.6H_2_O solution (22 g∙L^−1^) was added and after 6 min, 75 μL of a NaOH (0.8 M) solution. The absorbance was measured after 10 min of the reaction. The intrinsic absorption of samples was performed by replacing all the reagents (225 μL) with water. Catechin standard solutions were used for calibration. The results of the TFC were expressed as catechin equivalents (mg catechin/g dry leaf) and each extract was analyzed in quadruplicate.

#### 4.3.3. Total Antioxidant Capacity (TAC)

The total antioxidant capacity (TAC) was measured by means of the absorbance decrease at 734 mM of the diluted hydroalcoholic extract (1:50) due to the reduction of the ABTS^∙+^ radical [16]. Hence, 150 μL of the hydroalcoholic extracts and 150 μL of the ABTS^∙+^ standard solution (in acetate buffer, pH 4.6, 50 mM) were placed in each well of the 96-well plate. The absorbance reduction was monitored during 5 hours. The intrinsic absorption of samples was evaluated by replacing the ABTS^∙+^ radical solution with water. Trolox standard solutions were used for calibration. The TAC of the extracts was expressed as mM of Trolox per g of dry leaf and each extract was analyzed in quadruplicate.

### 4.4. Near Infrared Spectroscopy

Near infrared spectra of the air-dried powdered *Vaccinium* leaves were acquired on a Fourier-transform near-infrared spectrometer (FTLA 2000, ABB, Québec, state abbreviation, Canada) equipped with an indium-gallium-arsenide (InGaAs) detector in diffuse reflectance mode. Each spectrum resulted from an average of 64 scans with a resolution of 8 cm^−1^ in the wavenumber interval of 10000–4000 cm^−1^. Bomen-Grams software (version 7, ABB, Québec, Canada) was used to control the equipment. A total of five spectra *per* sample were acquired.

### 4.5. Data Analysis

Near infrared spectra were modelled by partial least squares (PLS) [30] to predict the total phenolic and flavonoid content as well as the total antioxidant capacity. Prior modelling, spectra were pre-processed with standard normal variate (SNV) and Savitzky-Golay filter (15 smoothing points, 2nd order polynomial and 1st derivative) [31] to remove baseline drifts and further mean centered. PLS models were optimized for the number of latent variables (LVs) and spectral region considering the leave-one-out cross-validation strategy. For the optimization of the spectral region, spectra were divided into five regions according to the Figure S1 which were tested separately or combined. The accuracy of the models was evaluated by means of the coefficient of determination of calibration, cross-validation and prediction (R^2^_C_, R^2^_CV_ and R^2^_P_), the root mean square error of calibration, cross-validation and prediction (RMSEC, RMSECV and RMSEP) and the range error ratio (RER). Calibration and prediction sets were randomly composed by 70% and 30% of the spectra, respectively. All chemometric models were performed in Matlab version 7.4 Release 2007a (MathWorks, Natick, MA, USA) and PLS Toolbox version 4.2.1 for Matlab (Eigenvector Research, Manson, WA, USA).

## 5. Conclusions

In this work the antioxidant activity of several blueberries cultivars leaves belonging to *V. corymbosum* and *V. ashei* by means of the TPC, TFC and TAC was determined. The considered cultivars exhibited very different antioxidant activities which also differ across the seasons (spring, fall and winter) and geographic regions considered. The knowledge about the antioxidant activity of such cultivars could be a valuable information allowing farmers to monetize the final product which is mostly wasted. Additionally, it was demonstrated that near infrared spectroscopy when coupled to appropriated chemometric models is a successful tool to predict the antioxidant activity of blueberries leaves cultivars. It should be noted that better prediction results were obtained considering leaves for a single harvest season (except for spring) and geographic origin. This result need to be taken into account when developing calibration models that could be applied to a global scale. However, when considering all the leaves (Set 1), the PLS models yielded RER values higher than 15 for TPC and TAC, and higher than 10 for TFC. These values reflect the accuracy and suitability of NIR spectroscopy for determining these parameters.

## Figures and Tables

**Table 1:** Total phenolic content (TPC), expressed in mg gallic acid/g dry leaf, obtained for all the cultivar leaves extracts harvested in the three geographic regions and three seasons across the vegetative stage.

Collecting Date		31th May 2017			1st September 2017	1st December 2017
Collecting Region		Region A	Region B	Region C	Region A	Region A
Cultivar	Legacy	106.2 ± 2.8	102.2 ± 3.2	-	64.3 ± 1.2	208.8 ± 3.6
	Ozarkblue	106.5 ± 2.5	-	-	78.9 ± 3.3	-
	Misty	157.6 ± 8.1	-	-	177.6 ± 6.2	176.5 ± 3.6
	Star	99.1 ± 2.4	-	-	-	170.1 ± 1.6
	Ochlochonee	127.3 ± 7.9	117.2 ± 5.2	-	217.9 ± 3.2	-
	Drapler	134.4 ± 3.8	55.2 ± 1.8	-	-	207.5 ± 7.9
	Bluecrop	127.8 ± 4.2	95.6 ± 2.9	-	120.4 ± 5.5	131.0 ± 2.1
	Duke	82.8 ± 11.6	74.3 ± 4.1	-	93.0 ± 1.3	118.6 ± 4.3
	Powderblue	161.2 ± 2.8	131.2 ± 4.8	-	-	219.7 ± 6.1
	O’neal	72.7 ±1.1	-	-	-	162.5 ± 1.1
	Chandler	123.8 ± 2.1	83.0 ± 9.4	-	166.5 ± 2.6	199.4 ± 5.9
	Bluejay	127.3 ± 5.4	-	-	-	-
	Camellia	104.2 ± 8.8	81.9 ± 12.5	-	69.3 ± 3.1	141.9 ± 2.6
	Goldtraube	114.2 ± 3.2	-	-	154.1 ±5.7	152.5 ± 3.5
	Titan	153.2 ± 2.6	-	-	40.8 ± 1.3	272.8 ± 4.0
	Huron	75.5 ± 2.2	-	-	39.6 ± 1.8	192.2 ± 3.9
	Aurora	110.9 ± 3.3	-	-	191.2 ± 6.3	227.4 ± 9.9
	Liberty	117.9 ± 14.8	-	-	157.3 ± 1.6	176.9 ± 6.0
	Bluegold	-	69.8 ± 3.9	-	-	-
	Elliott	-	-	-	-	199.0 ± 1.3
	Patriot	-	-	-	-	155.5 ± 5.2
	Biloxi	-	-	-	-	150.7 ± 3.9
	Alix blue	-	-	97.9 ± 5.9	-	-
	New hanover	-	-	92.7 ± 4.1	-	-
	Sunset blue	-	-	98.8 ± 5.0	-	-
	Gupton	-	-	96.6 ± 5.2	-	-
	Cipria	-	-	137.3 ± 4.4	-	-

Region A- Northern Coast; Region B- Northern Inland; Region C- South Inland.

**Table 2:** Total flavonoid content (TFC), expressed in mg catechin/g dry leaf, obtained for all the cultivar leaves extracts harvested in three geographic regions and three seasons across the vegetative stage.

Collecting Date		31th May 2017			1st September 2017	1st December 2017
Collecting Region		Region A	Region B	Region C	Region A	Region A
Cultivar	Legacy	181.3 ± 6.2	113.0 ± 2.9	-	60.5 ± 1.4	128.1 ± 3.7
	Ozarkblue	177.7 ± 2.9	-	-	59.2 ± 2.9	-
	Misty	269.1 ± 7.4	-	-	163.7 ± 10.0	143.0 ± 4.6
	Star	97.4 ± 3.9	-	-	-	125.1 ± 2.4
	Ochlochonee	181.1 ± 7.9	67.7 ± 2.9	-	192.7 ± 4.5	-
	Drapler	204.6 ± 20.6	71.0 ± 6.2	-	-	156.0 ± 2.3
	Bluecrop	198.1 ± 9.8	73.3 ± 3.9	-	96.4 ± 4.0	86.4 ± 2.3
	Duke	79.6 ± 9.7	108.9 ± 5.1	-	71.3 ± 2.2	87.5 ± 3.2
	Powderblue	248.8 ± 20.4	72.2 ± 5.1	-	-	189.1 ± 6.0
	O’neal	111.3 ± 4.4	-	-	-	106.2 ± 2.4
	Chandler	119.2 ± 3.5	74.9 ± 4.0	-	133.9 ± 4.4	156.7 ± 7.6
	Bluejay	184.5 ± 5.4	-	-	-	-
	Camellia	79.0 ± 6.5	45.2 ± 2.3	-	47.3 ± 2.0	101.4 ± 2.2
	Goldtraube	110.7 ± 4.9	-	-	115.1 ± 4.4	118.1 ± 5.7
	Titan	244.2 ± 7.5	-	-	47.5 ± 4.6	242.5 ± 6.9
	Huron	103.0 ± 5.6	-	-	41.2 ± 4.8	133.8 ± 6.0
	Aurora	103.4 ± 4.2	-	-	141.0 ± 5.9	150.8 ± 6.6
	Liberty	78.8 ± 5.8	-	-	50.0 ± 4.6	126.4 ± 4.2
	Bluegold	-	94.2 ± 3.5	-	-	-
	Elliott	-	-	-	-	131.9 ± 4.1
	Patriot	-	-	-	-	89.6 ± 5.6
	Biloxi	-	-	-	-	96.0 ± 3.1
	Alix blue	-	-	88.8 ± 5.2	-	-
	New hanover	-	-	96.2 ± 6.9	-	-
	Sunset blue	-	-	92.5 ± 8.4	-	-
	Gupton	-	-	97.3 ± 4.8	-	-
	Cipria	-	-	129.2 ± 4.0	-	-

Region A- Northern Coast; Region B- Northern Inland; Region C- South Inland.

**Table 3:** Total antioxidant capacity (TAC), expressed in mM of Trolox/g dry leaf, obtained for all the cultivar leaves extracts harvested in the three geographic regions and three seasons across the vegetative stage.

Collecting Date		31th May 2017			1st September 2017	1st December 2017
Collecting Region		Region A	Region B	Region C	Region A	Region A
Cultivar	Legacy	53.8 ± 1.2	56.3 ± 1.0	-	34.4 ± 1.3	81.4 ± 0.7
	Ozarkblue	50.3 ± 0.8	-	-	43.1 ± 2.7	-
	Misty	63.7 ± 2.0	-	-	99.6 ± 3.3	72.6 ± 1.9
	Star	46.5 ± 1.5	-	-	-	76.4 ± 0.9
	Ochlochonee	62.8 ± 0.8	56.3 ± 0.9	-	101.1 ± 1.4	-
	Drapler	60.8 ± 1.7	34.5 ± 2.2	-	-	107.8 ± 3.8
	Bluecrop	57.0 ± 1.2	51.7 ± 1.5	-	55.6 ± 1.8	61.1 ± 2.2
	Duke	39.3 ± 1.5	40.0 ± 1.3	-	47.6 ± 1.7	61.0 ± 1.8
	Powderblue	65.5 ± 1.0	68.2 ± 2.1	-	-	115.8 ± 3.5
	O’neal	40.3 ± 0.3	-	-	-	66.7 ± 0.9
	Chandler	55.8 ± 0.8	43.0 ± 3.7	-	92.1 ± 0.9	107.3 ± 0.9
	Bluejay	59.1 ± 1.7	-	-	-	-
	Camellia	50.8 ± 0.9	44.5 ± 4.0	-	39.2 ± 1.2	62.2 ± 1.5
	Goldtraube	50.2 ± 3.3	-	-	86.2 ± 1.2	70.6 ± 2.1
	Titan	63.1 ± 1.3	-	-	23.2 ± 1.1	124.8 ± 2.5
	Huron	41.7 ± 1.3	-	-	22.6 ± 0.7	113.4 ± 3.1
	Aurora	54.9 ± 3.9	-	-	100.0 ± 2.6	120.0 ± 1.2
	Liberty	61.9 ± 4.4	-	-	23.3 ± 1.6	73.3 ± 1.7
	Bluegold	-	44.7 ± 2.5	-	-	-
	Elliott	-	-	-	-	78.2 ± 0.9
	Patriot	-	-	-	-	64.5 ± 1.7
	Biloxi	-	-	-	-	62.2 ± 1.5
	Alix blue	-	-	42.9 ± 1.2	-	-
	New hanover	-	-	43.0 ± 1.9	-	-
	Sunset blue	-	-	48.6 ± 4.6	-	-
	Gupton	-	-	46.4 ± 3.8	-	-
	Cipria	-	-	56.0 ± 5.0	-	-

Region A- Northern Coast; Region B- Northern Inland; Region C- South Inland.

**Table 4:** Figures of merit obtained from the PLS models developed to predict the total phenolic and flavonoids content and total antioxidant capacity of *Vaccinium* cultivar leaves by NIR spectroscopy.

Parameter	Data Set	Spectral Range	LVs	RMSEC	R^2^_C_	RMSECV	R^2^_CV_	RMSEP	R^2^_P_	RER
Total Phenolic Content	1	6315–5390 and 4925–4073	5	12.3	0.94	13.1	0.93	12.6	0.93	18.5
	2	6315–5390 and 4925–4073	7	12.6	0.94	13.9	0.92	13.5	0.94	17.3
	3	6315–5390 and 4925–4073	5	8.0	0.91	9.1	0.88	8.8	0.89	12.0
	4	6315–5390 and 4925–4073	5	4.7	0.97	5.8	0.95	5.7	0.94	15.0
	5	6315–5390 and 4925–4073	5	6.3	0.99	7.9	0.98	6.9	0.99	25.5
	6	6315–5390 and 4925–4073	6	8.4	0.95	10.2	0.93	8.8	0.93	17.4
Total Flavonoids Content	1	6315–5390 and 4925–4073	9	16.4	0.86	18.1	0.83	16.6	0.88	12.1
	2	6315–5390 and 4925–4073	9	12.1	0.95	13.9	0.93	12.5	0.94	18.3
	3	6315–5390 and 4925–4073	9	17.3	0.89	21.8	0.83	18.7	0.84	12.0
	4	6315–5390 and 4925–4073	9	15.5	0.92	22.8	0.82	16.8	0.94	11.4
	5	6315–5390 and 4925–4073	6	5.8	0.98	7.4	0.97	6.7	0.98	22.7
	6	6315–5390 and 4925–4073	6	7.2	0.97	8.6	0.95	7.3	0.95	21.4
Total Antioxidant Capacity	1	6315–5390 and 4925–4073	7	6.2	0.92	6.8	0.91	6.4	0.93	16.0
	2	6315–5390 and 4925–4073	7	6.5	0.93	7.1	0.92	7.1	0.92	14.3
	3	6315–5390 and 4925–4073	7	3.0	0.90	3.5	0.86	3.0	0.88	11.2
	4	6315–5390 and 4925–4073	7	1.9	0.95	2.4	0.92	1.9	0.94	13.5
	5	6315–5390 and 4925–4073	7	2.3	0.99	3.8	0.98	2.9	0.99	26.8
	6	6315–5390 and 4925–4073	6	3.5	0.98	4.5	0.96	3.7	0.98	17.4

* All data were pre-processed with SavGol(15,2,1) and SNV and further mean centered. Set 1- all the leaves (May/September/December, RA/RB/RC); Set 2- leaves collected from a single region (May/September/December, RA); Set 3- leaves collected in May (RA/RB/RC); Set 4- leaves collected in May (RA); Set 5- leaves collected in September (RA) and Set 6- leaves collected in December (RA). RER-Δy/RMSEP; RA- Northern Coast; RB- Northern Inland; RC- South Inland

**Table 5:** *Vaccinium* spp. cultivar leaves included in this study.

Species	Cultivar	Nº	May				June				July				August				September				October				November				December			
			1	2	3	4	1	2	3	4	1	2	3	4	1	2	3	4	1	2	3	4	1	2	3	4	1	2	3	4	1	2	3	4
*V. corymbosum*	Biloxi	1				X																												
	New hanover	1				X																												
	Camellia	2				X													X												X			
	O’neal	1				X																									X			
	Misty	1				X													X												X			
	Alix blue	1				X																												
	Star	1				X																									X			
	Gupton	1				X																												
	Cipria	1				X																												
	Goldtraube	1				X													X												X			
	Legacy	2				X													X												X			
	Ozarkblue	1				X													X															
	Bluegold	1				X																												
	Draper	2				X																									X			
	Chandler	2				X													X												X			
	Liberty	1				X													X												X			
	Bluecrop	2				X													X												X			
	Sunset blue	1				X																												
	Duke	2				X													X															
	Patriot	1				X																									X			
	Bluejay	1				X																												
	Huron	1				X													X												X			
	Aurora	1				X													X												X			
	Elliott	1				X																									X			
*V. ashei*	Titan	1				X													X												X			
	Ochlochonee	2				X													X															
	Powderblue	2				X																									X			

Nº- number of plants per cultivar included in the study; X- Leaves harvest month and week. Shaded cells denoted the presence of berries in the different plants.

## Supplementary Materials

The Supplementary Materials are available online.
