# Supplementary material for: Antioxidant Activity of Blueberry (Vaccinium spp.) Cultivar Leaves: Differences across the Vegetative Stage and the Application of Near Infrared Spectroscopy
Source: Molecules. 2019 Oct 29;24(21):3900. doi: 10.3390/molecules24213900 (PMC6864474; doi:10.3390/molecules24213900)
Supplement: Supplementary file 1 [file molecules-24-03900-s001.pdf]

**Table S1.** Statistical data analysis for the total phenolic content (TPC) of *Vaccinium* cultivars collected in Spring, region A: Northern Coast. Red cells correspond to significantly different values ($p < 0.05$) and green ones to statistically equal values ($p > 0.05$).

| | Leg | Oz | Mis | Str | Och | Dra | Bluc | Duk | Pow | One | Cha | Bluj | Cam | Gol | Tit | Hur | Aur | Lib |
|---|---|---|---|---|---|---|---|---|---|---|---|---|---|---|---|---|---|---|
| Leg | | | | | | | | | | | | | | | | | | |
| Oz | | | | | | | | | | | | | | | | | | |
| Mis | | | | | | | | | | | | | | | | | | |
| Str | | | | | | | | | | | | | | | | | | |
| Och | | | | | | | | | | | | | | | | | | |
| Dra | | | | | | | | | | | | | | | | | | |
| Bluc | | | | | | | | | | | | | | | | | | |
| Duk | | | | | | | | | | | | | | | | | | |
| Pow | | | | | | | | | | | | | | | | | | |
| One | | | | | | | | | | | | | | | | | | |
| Cha | | | | | | | | | | | | | | | | | | |
| Bluj | | | | | | | | | | | | | | | | | | |
| Cam | | | | | | | | | | | | | | | | | | |
| Gol | | | | | | | | | | | | | | | | | | |
| Tit | | | | | | | | | | | | | | | | | | |
| Hur | | | | | | | | | | | | | | | | | | |
| Aur | | | | | | | | | | | | | | | | | | |
| Lib | | | | | | | | | | | | | | | | | | |

Leg: Legacy; Oz: Ozarkblue; Mis: Misty; Str: Star; Och: Ochlochonee; Dra: Drapler; Bluc: Bluecrop; Duk: Duke; Pow: Powderblue; One: O'neal; Cha: Chandler; Bluj: Bluejay; Cam: Camellia: Gol: Goldtraube; Tit: Titan; Hur: Huron; Aur: Aurora; Lib: Liberty

**Table S2.** Statistical data analysis for the total phenolic content (TPC) of *Vaccinium* cultivars collected in Fall, region A: Northern Inland. Red cells correspond to significantly different values ($p < 0.05$) and green ones to statistically equal values ($p > 0.05$).

| | Leg | Oz | Mis | Och | Bluc | Duk | Cha | Cam | Gol | Tit | Hur | Aur | Lib |
|---|---|---|---|---|---|---|---|---|---|---|---|---|---|
| Leg | | | | | | | | | | | | | |
| Oz | | | | | | | | | | | | | |
| Mis | | | | | | | | | | | | | |
| Och | | | | | | | | | | | | | |
| Bluc | | | | | | | | | | | | | |
| Duk | | | | | | | | | | | | | |
| Cha | | | | | | | | | | | | | |
| Cam | | | | | | | | | | | | | |
| Gol | | | | | | | | | | | | | |
| Tit | | | | | | | | | | | | | |
| Hur | | | | | | | | | | | | | |
| Aur | | | | | | | | | | | | | |
| Lib | | | | | | | | | | | | | |

Leg: Legacy; Oz: Ozarkblue; Mis: Misty; Och: Ochlochonee; Bluc: Bluecrop; Duk: Duke; Cha: Chandler; Cam: Camellia: Gol: Goldtraube; Tit: Titan; Hur: Huron; Aur: Aurora; Lib: Liberty

**Table S3.** Statistical data analysis for the total phenolic content (TPC) of *Vaccinium* cultivars collected in Winter, region A: South Inland. Red cells correspond to significantly different values ($p < 0.05$) and green ones to statistically equal values ($p > 0.05$).

| | Leg | Mis | Str | Dra | Bluc | Duk | Pow | One | Cha | Cam | Gol | Tit | Hur | Aur | Lib | Ell | Pat | Bil |
|---|---|---|---|---|---|---|---|---|---|---|---|---|---|---|---|---|---|---|
| Leg | | | | | | | | | | | | | | | | | | |
| Mis | | | | | | | | | | | | | | | | | | |
| Str | | | | | | | | | | | | | | | | | | |
| Dra | | | | | | | | | | | | | | | | | | |
| Bluc | | | | | | | | | | | | | | | | | | |
| Duk | | | | | | | | | | | | | | | | | | |
| Pow | | | | | | | | | | | | | | | | | | |
| One | | | | | | | | | | | | | | | | | | |
| Cha | | | | | | | | | | | | | | | | | | |
| Cam | | | | | | | | | | | | | | | | | | |
| Gol | | | | | | | | | | | | | | | | | | |
| Tit | | | | | | | | | | | | | | | | | | |
| Hur | | | | | | | | | | | | | | | | | | |
| Aur | | | | | | | | | | | | | | | | | | |
| Lib | | | | | | | | | | | | | | | | | | |
| Ell | | | | | | | | | | | | | | | | | | |
| Pat | | | | | | | | | | | | | | | | | | |
| Bil | | | | | | | | | | | | | | | | | | |

Leg: Legacy; Mis: Misty; Str: Star; Dra: Drapler; Bluc: Bluecrop; Duk: Duke; Pow: Powderblue; One: O'neal; Cha: Chandler; Cam: Camellia: Gol: Goldtraube; Tit: Titan; Hur: Huron; Aur: Aurora; Lib: Liberty; Ell: Elliot; Pat: Patriot; Bil: Biloxi

**Table S4.** Statistical data analysis for the total phenolic content (TPC) of each *Vaccinium* cultivar collected in Spring, Fall and Winter (Region A: Northern Coast). Red cells correspond to significantly different values ($p < 0.05$) and green ones to statistically equal values ($p > 0.05$).

| | Fall | Winter |
|---|---|---|
| Leg (Spring) | | |
| Leg (Fall) | | |
| Oz (Spring) | | --- |
| Oz (Fall) | | --- |
| Mis (Spring) | | |
| Mis (Fall) | | |
| Str (Spring) | --- | |
| Str (Fall) | | --- |
| Och (Spring) | | --- |
| Och (Fall) | | --- |
| Dra (Spring) | --- | |
| Dra (Fall) | | --- |
| Bluc (Spring) | | |
| Bluc (Fall) | | |
| Duk (Spring) | | |
| Duk (Fall) | | |
| Pow (Spring) | --- | |
| Pow (Fall) | | --- |

| | Fall | Winter |
|---|---|---|
| One (Spring) | --- | |
| One (Fall) | | --- |
| Cha (Spring) | | |
| Cha (Fall) | | |
| Cam (Spring) | | |
| Cam (Fall) | | |
| Gol (Spring) | | |
| Gol (Fall) | | |
| Tit (Spring) | | |
| Tit (Fall) | | |
| Hur (Spring) | | |
| Hur (Fall) | | |
| Aur (Spring) | | |
| Aur (Fall) | | |
| Lib (Spring) | | |
| Lib (Fall) | | |

Leg: Legacy; Oz: Ozarkblue; Mis: Misty; Str: Star; Och: Ochlochonee; Dra: Drapler; Bluc: Bluecrop; Duk: Duke; Pow: Powderblue; One: O'neal; Cha: Chandler; Bluj: Bluejay; Cam: Camellia: Gol: Goldtraube; Tit: Titan; Hur: Huron; Aur: Aurora; Lib: Liberty

**Table S5.** Statistical data analysis for the total phenolic content (TPC) of *Vaccinium* cultivars collected in Spring, region B: Northern Inland. Red cells correspond to significantly different values ($p < 0.05$) and green ones to statistically equal values ($p > 0.05$).

| | Leg | Och | Dra | Bluc | Duk | Pow | Cha | Cam | Blug |
|---|---|---|---|---|---|---|---|---|---|
| Leg | | | | | | | | | |
| Och | | | | | | | | | |
| Dra | | | | | | | | | |
| Bluc | | | | | | | | | |
| Duk | | | | | | | | | |
| Pow | | | | | | | | | |
| Cha | | | | | | | | | |
| Cam | | | | | | | | | |
| Blug | | | | | | | | | |

Leg: Legacy; Och: Ochlochonee; Dra: Drapler; Bluc: Bluecrop; Duk: Duke; Pow: Powderblue; Cha: Chandler; Cam: Camellia: Blug: Bluegold

**Table S6.** Statistical data analysis for the total phenolic content (TPC) of *Vaccinium* cultivars collected in Spring, region C: South Inland. Red cells correspond to significantly different values ($p < 0.05$) and green ones to statistically equal values ($p > 0.05$).

| | Alx | Neh | Sun | Gup | Cip |
|---|---|---|---|---|---|
| Alx | | | | | |
| Neh | | | | | |
| Sun | | | | | |
| Gup | | | | | |
| Cip | | | | | |

Alx: Alix blue; Neh: New hanover; Sun: Sunset blue; Gup: Gupton; Cip: Cipria

**Table S7.** Statistical data analysis for the total flavonoid content (TFC) of *Vaccinium* cultivars collected in Spring, region A: Northern Coast. Red cells correspond to significantly different values ($p < 0.05$) and green ones to statistically equal values ($p > 0.05$).

| | Leg | Oz | Mis | Str | Och | Dra | Bluc | Duk | Pow | One | Cha | Bluj | Cam | Gol | Tit | Hur | Aur | Lib |
|---|---|---|---|---|---|---|---|---|---|---|---|---|---|---|---|---|---|---|
| Leg | | | | | | | | | | | | | | | | | | |
| Oz | | | | | | | | | | | | | | | | | | |
| Mis | | | | | | | | | | | | | | | | | | |
| Str | | | | | | | | | | | | | | | | | | |
| Och | | | | | | | | | | | | | | | | | | |
| Dra | | | | | | | | | | | | | | | | | | |
| Bluc | | | | | | | | | | | | | | | | | | |
| Duk | | | | | | | | | | | | | | | | | | |
| Pow | | | | | | | | | | | | | | | | | | |
| One | | | | | | | | | | | | | | | | | | |
| Cha | | | | | | | | | | | | | | | | | | |
| Bluj | | | | | | | | | | | | | | | | | | |
| Cam | | | | | | | | | | | | | | | | | | |
| Gol | | | | | | | | | | | | | | | | | | |
| Tit | | | | | | | | | | | | | | | | | | |
| Hur | | | | | | | | | | | | | | | | | | |
| Aur | | | | | | | | | | | | | | | | | | |
| Lib | | | | | | | | | | | | | | | | | | |

Leg: Legacy; Oz: Ozarkblue; Mis: Misty; Str: Star; Och: Ochlochonee; Dra: Drapler; Bluc: Bluecrop; Duk: Duke; Pow: Powderblue; One: O'neal; Cha: Chandler; Bluj: Bluejay; Cam: Camellia: Gol: Goldtraube; Tit: Titan; Hur: Huron; Aur: Aurora; Lib: Liberty

**Table S8.** Statistical data analysis for the total flavonoid content (TFC) of *Vaccinium* cultivars collected in Fall, region A: Northern Inland. Red cells correspond to significantly different values ($p < 0.05$) and green ones to statistically equal values ($p > 0.05$).

| | Leg | Oz | Mis | Och | Bluc | Duk | Cha | Cam | Gol | Tit | Hur | Aur | Lib |
|---|---|---|---|---|---|---|---|---|---|---|---|---|---|
| Leg | | | | | | | | | | | | | |
| Oz | | | | | | | | | | | | | |
| Mis | | | | | | | | | | | | | |
| Och | | | | | | | | | | | | | |
| Bluc | | | | | | | | | | | | | |
| Duk | | | | | | | | | | | | | |
| Cha | | | | | | | | | | | | | |
| Cam | | | | | | | | | | | | | |
| Gol | | | | | | | | | | | | | |
| Tit | | | | | | | | | | | | | |
| Hur | | | | | | | | | | | | | |
| Aur | | | | | | | | | | | | | |
| Lib | | | | | | | | | | | | | |

Leg: Legacy; Oz: Ozarkblue; Mis: Misty; Och: Ochlochonee; Bluc: Bluecrop; Duk: Duke; Cha: Chandler; Cam: Camellia: Gol: Goldtraube; Tit: Titan; Hur: Huron; Aur: Aurora; Lib: Liberty

**Table S9.** Statistical data analysis for the total flavonoid content (TFC) of *Vaccinium* cultivars collected in Winter, region A: South Inland. Red cells correspond to significantly different values ($p < 0.05$) and green ones to statistically equal values ($p > 0.05$).

| | Leg | Mis | Str | Dra | Bluc | Duk | Pow | One | Cha | Cam | Gol | Tit | Hur | Aur | Lib | Ell | Pat | Bil |
|---|---|---|---|---|---|---|---|---|---|---|---|---|---|---|---|---|---|---|
| Leg | | | | | | | | | | | | | | | | | | |
| Mis | | | | | | | | | | | | | | | | | | |
| Str | | | | | | | | | | | | | | | | | | |
| Dra | | | | | | | | | | | | | | | | | | |
| Bluc | | | | | | | | | | | | | | | | | | |
| Duk | | | | | | | | | | | | | | | | | | |
| Pow | | | | | | | | | | | | | | | | | | |
| One | | | | | | | | | | | | | | | | | | |
| Cha | | | | | | | | | | | | | | | | | | |
| Cam | | | | | | | | | | | | | | | | | | |
| Gol | | | | | | | | | | | | | | | | | | |
| Tit | | | | | | | | | | | | | | | | | | |
| Hur | | | | | | | | | | | | | | | | | | |
| Aur | | | | | | | | | | | | | | | | | | |
| Lib | | | | | | | | | | | | | | | | | | |
| Ell | | | | | | | | | | | | | | | | | | |
| Pat | | | | | | | | | | | | | | | | | | |
| Bil | | | | | | | | | | | | | | | | | | |

Leg: Legacy; Mis: Misty; Str: Star; Dra: Drapler; Bluc: Bluecrop; Duk: Duke; Pow: Powderblue; One: O'neal; Cha: Chandler; Cam: Camellia: Gol: Goldtraube; Tit: Titan; Hur: Huron; Aur: Aurora; Lib: Liberty; Ell: Elliot; Pat: Patriot; Bil: Biloxi

**Table S10.** Statistical data analysis for the total flavonoid content (TFC) of each *Vaccinium* cultivar collected in Spring, Fall and Winter (Region A: Northern Coast). Red cells correspond to significantly different values ($p < 0.05$) and green ones to statistically equal values ($p > 0.05$).

| | Fall | Winter |
|---|---|---|
| Leg (Spring) | | |
| Leg (Fall) | | |
| Oz (Spring) | | --- |
| Oz (Fall) | | --- |
| Mis (Spring) | | |
| Mis (Fall) | | |
| Str (Spring) | --- | |
| Str (Fall) | | --- |
| Och (Spring) | | --- |
| Och (Fall) | | --- |
| Dra (Spring) | --- | |
| Dra (Fall) | | --- |
| Bluc (Spring) | | |
| Bluc (Fall) | | |
| Duk (Spring) | | |
| Duk (Fall) | | |
| Pow (Spring) | --- | |
| Pow (Fall) | | --- |

| | Fall | Winter |
|---|---|---|
| One (Spring) | --- | |
| One (Fall) | | --- |
| Cha (Spring) | | |
| Cha (Fall) | | |
| Cam (Spring) | | |
| Cam (Fall) | | |
| Gol (Spring) | | |
| Gol (Fall) | | |
| Tit (Spring) | | |
| Tit (Fall) | | |
| Hur (Spring) | | |
| Hur (Fall) | | |
| Aur (Spring) | | |
| Aur (Fall) | | |
| Lib (Spring) | | |
| Lib (Fall) | | |

Leg: Legacy; Oz: Ozarkblue; Mis: Misty; Str: Star; Och: Ochlochonee; Dra: Drapler; Bluc: Bluecrop; Duk: Duke; Pow: Powderblue; One: O'neal; Cha: Chandler; Cam: Camellia: Gol: Goldtraube; Tit: Titan; Hur: Huron; Aur: Aurora; Lib: Liberty

**Table S11.** Statistical data analysis for the total flavonoid content (TFC) of *Vaccinium* cultivars collected in Spring, region B: Northern Inland. Red cells correspond to significantly different values ($p < 0.05$) and green ones to statistically equal values ($p > 0.05$).

| | Leg | Och | Dra | Bluc | Duk | Pow | Cha | Cam | Blug |
|---|---|---|---|---|---|---|---|---|---|
| Leg | | | | | | | | | |
| Och | | | | | | | | | |
| Dra | | | | | | | | | |
| Bluc | | | | | | | | | |
| Duk | | | | | | | | | |
| Pow | | | | | | | | | |
| Cha | | | | | | | | | |
| Cam | | | | | | | | | |
| Blug | | | | | | | | | |

Leg: Legacy; Och: Ochlochonee; Dra: Drapler; Bluc: Bluecrop; Duk: Duke; Pow: Powderblue; Cha: Chandler; Cam: Camellia: Blug: Bluegold

**Table S12.** Statistical data analysis for the total flavonoid content (TFC) of *Vaccinium* cultivars collected in Spring, region C: South Inland. Red cells correspond to significantly different values ($p < 0.05$) and green ones to statistically equal values ($p > 0.05$).

| | Alx | Neh | Sun | Gup | Cip |
|---|---|---|---|---|---|
| Alx | | | | | |
| Neh | | | | | |
| Sun | | | | | |
| Gup | | | | | |
| Cip | | | | | |

Alx: Alix blue; Neh: New hanover; Sun: Sunset blue; Gup: Gupton; Cip: Cipria

**Table S13.** Statistical data analysis for the total antioxidant activity (TAC) of *Vaccinium* cultivars collected in Spring, region A: Northern Coast. Red cells correspond to significantly different values ($p < 0.05$) and green ones to statistically equal values ($p > 0.05$).

| | Leg | Oz | Mis | Str | Och | Dra | Bluc | Duk | Pow | One | Cha | Bluj | Cam | Gol | Tit | Hur | Aur | Lib |
|---|---|---|---|---|---|---|---|---|---|---|---|---|---|---|---|---|---|---|
| Leg | | | | | | | | | | | | | | | | | | |
| Oz | | | | | | | | | | | | | | | | | | |
| Mis | | | | | | | | | | | | | | | | | | |
| Str | | | | | | | | | | | | | | | | | | |
| Och | | | | | | | | | | | | | | | | | | |
| Dra | | | | | | | | | | | | | | | | | | |
| Bluc | | | | | | | | | | | | | | | | | | |
| Duk | | | | | | | | | | | | | | | | | | |
| Pow | | | | | | | | | | | | | | | | | | |
| One | | | | | | | | | | | | | | | | | | |
| Cha | | | | | | | | | | | | | | | | | | |
| Bluj | | | | | | | | | | | | | | | | | | |
| Cam | | | | | | | | | | | | | | | | | | |
| Gol | | | | | | | | | | | | | | | | | | |
| Tit | | | | | | | | | | | | | | | | | | |
| Hur | | | | | | | | | | | | | | | | | | |
| Aur | | | | | | | | | | | | | | | | | | |
| Lib | | | | | | | | | | | | | | | | | | |

Leg: Legacy; Oz: Ozarkblue; Mis: Misty; Str: Star; Och: Ochlochonee; Dra: Drapler; Bluc: Bluecrop; Duk: Duke; Pow: Powderblue; One: O'neal; Cha: Chandler; Bluj: Bluejay; Cam: Camellia: Gol: Goldtraube; Tit: Titan; Hur: Huron; Aur: Aurora; Lib: Liberty

**Table S14.** Statistical data analysis for the total antioxidant activity (TAC) of *Vaccinium* cultivars collected in Fall, region A: Northern Inland. Red cells correspond to significantly different values ($p< 0.05$) and green ones to statistically equal values ($p> 0.05$).

| | Leg | Oz | Mis | Och | Bluc | Duk | Cha | Cam | Gol | Tit | Hur | Aur | Lib |
|---|---|---|---|---|---|---|---|---|---|---|---|---|---|
| Leg | | | | | | | | | | | | | |
| Oz | | | | | | | | | | | | | |
| Mis | | | | | | | | | | | | | |
| Och | | | | | | | | | | | | | |
| Bluc | | | | | | | | | | | | | |
| Duk | | | | | | | | | | | | | |
| Cha | | | | | | | | | | | | | |
| Cam | | | | | | | | | | | | | |
| Gol | | | | | | | | | | | | | |
| Tit | | | | | | | | | | | | | |
| Hur | | | | | | | | | | | | | |
| Aur | | | | | | | | | | | | | |
| Lib | | | | | | | | | | | | | |

Leg: Legacy; Oz: Ozarkblue; Mis: Misty; Och: Ochlochonee; Bluc: Bluecrop; Duk: Duke; Cha: Chandler; Cam: Camellia: Gol: Goldtraube; Tit: Titan; Hur: Huron; Aur: Aurora; Lib: Liberty

**Table S15.** Statistical data analysis for the total antioxidant activity (TAC) of *Vaccinium* cultivars collected in Winter, region A: South Inland. Red cells correspond to significantly different values ($p < 0.05$) and green ones to statistically equal values ($p > 0.05$).

| | Leg | Mis | Str | Dra | Bluc | Duk | Pow | One | Cha | Cam | Gol | Tit | Hur | Aur | Lib | Ell | Pat | Bil |
|---|---|---|---|---|---|---|---|---|---|---|---|---|---|---|---|---|---|---|
| Leg | | | | | | | | | | | | | | | | | | |
| Mis | | | | | | | | | | | | | | | | | | |
| Str | | | | | | | | | | | | | | | | | | |
| Dra | | | | | | | | | | | | | | | | | | |
| Bluc | | | | | | | | | | | | | | | | | | |
| Duk | | | | | | | | | | | | | | | | | | |
| Pow | | | | | | | | | | | | | | | | | | |
| One | | | | | | | | | | | | | | | | | | |
| Cha | | | | | | | | | | | | | | | | | | |
| Cam | | | | | | | | | | | | | | | | | | |
| Gol | | | | | | | | | | | | | | | | | | |
| Tit | | | | | | | | | | | | | | | | | | |
| Hur | | | | | | | | | | | | | | | | | | |
| Aur | | | | | | | | | | | | | | | | | | |
| Lib | | | | | | | | | | | | | | | | | | |
| Ell | | | | | | | | | | | | | | | | | | |
| Pat | | | | | | | | | | | | | | | | | | |
| Bil | | | | | | | | | | | | | | | | | | |

Leg: Legacy; Mis: Misty; Str: Star; Dra: Drapler; Bluc: Bluecrop; Duk: Duke; Pow: Powderblue; One: O'neal; Cha: Chandler; Cam: Camellia: Gol: Goldtraube; Tit: Titan; Hur: Huron; Aur: Aurora; Lib: Liberty; Ell: Elliot; Pat: Patriot; Bil: Biloxi

**Table S16.** Statistical data analysis for the total antioxidant activity (TAC) of each *Vaccinium* cultivar collected in Spring, Fall and Winter (Region A: Northern Coast). Red cells correspond to significantly different values ($p < 0.05$) and green ones to statistically equal values ($p > 0.05$).

| | Fall | Winter |
|---|---|---|
| Leg (Spring) | | |
| Leg (Fall) | | |
| Oz (Spring) | | --- |
| Oz (Fall) | | --- |
| Mis (Spring) | | |
| Mis (Fall) | | |
| Str (Spring) | --- | |
| Str (Fall) | | --- |
| Och (Spring) | | --- |
| Och (Fall) | | --- |
| Dra (Spring) | --- | |
| Dra (Fall) | | --- |
| Bluc (Spring) | | |
| Bluc (Fall) | | |
| Duk (Spring) | | |
| Duk (Fall) | | |
| Pow (Spring) | --- | |
| Pow (Fall) | | --- |

| | Fall | Winter |
|---|---|---|
| One (Spring) | --- | |
| One (Fall) | | --- |
| Cha (Spring) | | |
| Cha (Fall) | | |
| Cam (Spring) | | |
| Cam (Fall) | | |
| Gol (Spring) | | |
| Gol (Fall) | | |
| Tit (Spring) | | |
| Tit (Fall) | | |
| Hur (Spring) | | |
| Hur (Fall) | | |
| Aur (Spring) | | |
| Aur (Fall) | | |
| Lib (Spring) | | |
| Lib (Fall) | | |

Leg: Legacy; Oz: Ozarkblue; Mis: Misty; Str: Star; Och: Ochlochonee; Dra: Drapler; Bluc: Bluecrop; Duk: Duke; Pow: Powderblue; One: O’neal; Cha: Chandler; Cam: Camellia: Gol: Goldtraube; Tit: Titan; Hur: Huron; Aur: Aurora; Lib: Liberty

**Table S17.** Statistical data analysis for the total antioxidant activity (TAC) of *Vaccinium* cultivars collected in Spring, region B: Northern Inland. Red cells correspond to significantly different values ($p < 0.05$) and green ones to statistically equal values ($p > 0.05$).

| | Leg | Och | Dra | Bluc | Duk | Pow | Cha | Cam | Blug |
|---|---|---|---|---|---|---|---|---|---|
| Leg | | | | | | | | | |
| Och | | | | | | | | | |
| Dra | | | | | | | | | |
| Bluc | | | | | | | | | |
| Duk | | | | | | | | | |
| Pow | | | | | | | | | |
| Cha | | | | | | | | | |
| Cam | | | | | | | | | |
| Blug | | | | | | | | | |

Leg: Legacy; Och: Ochlochonee; Dra: Drapler; Bluc: Bluecrop; Duk: Duke; Pow: Powderblue; Cha: Chandler; Cam: Camellia: Blug: Bluegold

**Table S18.** Statistical data analysis for the total antioxidant activity (TAC) of *Vaccinium* cultivars collected in Spring, region C: South Inland. Red cells correspond to significantly different values ($p < 0.05$) and green ones to statistically equal values ($p > 0.05$).

| | Alx | Neh | Sun | Gup | Cip |
|---|---|---|---|---|---|
| Alx | | | | | |
| Neh | | | | | |
| Sun | | | | | |
| Gup | | | | | |
| Cip | | | | | |

Alx: Alix blue; Neh: New hanover; Sun: Sunset blue; Gup: Gupton; Cip: Cipria
